# Chimeric Antigen Receptor-T Cell and Oncolytic Viral Therapies for Gastric Cancer and Peritoneal Carcinomatosis of Gastric Origin: Path to Improving Combination Strategies

**DOI:** 10.3390/cancers15235661

**Published:** 2023-11-30

**Authors:** Courtney Chen, Audrey Jung, Annie Yang, Isabel Monroy, Zhifang Zhang, Shyambabu Chaurasiya, Supriya Deshpande, Saul Priceman, Yuman Fong, Anthony K. Park, Yanghee Woo

**Affiliations:** 1 Department of Surgery, City of Hope, Duarte, CA 91010, USA; cochen@coh.org (C.C.); audreysjung@gmail.com (A.J.); ayang@coh.org (A.Y.); zhzhang@coh.org (Z.Z.); schaurasiya@coh.org (S.C.); sudeshpande@coh.org (S.D.); yfong@coh.org (Y.F.); 2 Department of Hematology and Hematopoietic Cell Transplantation, City of Hope, Duarte, CA 91010, USA; imonroy@coh.org (I.M.); spriceman@coh.org (S.P.); 3 Cancer Immunotherapeutics Program, Beckman Research Institute, City of Hope, Duarte, CA 91010, USA

**Keywords:** CAR T cells, oncolytic virus, gastric cancer, peritoneal carcinomatosis, combination therapy

## Abstract

**Simple Summary:**

Gastric cancer (GC) peritoneal carcinomatosis (PC) is treatment refractory, and GCPC patient survival remains poor despite the use of better systemic and regional therapeutic strategies. Alternative approaches are required to overcome the distinct barriers to improve outcomes in GCPC patients. Here, we review recent developments in CAR T cell therapy and oncolytic virotherapy in GCPC and present the exciting potential of a combination immunotherapeutic approach.

**Abstract:**

Precision immune oncology capitalizes on identifying and targeting tumor-specific antigens to enhance anti-tumor immunity and improve the treatment outcomes of solid tumors. Gastric cancer (GC) is a molecularly heterogeneous disease where monoclonal antibodies against human epidermal growth factor receptor 2 (HER2), vascular endothelial growth factor (VEGF), and programmed cell death 1 (PD-1) combined with systemic chemotherapy have improved survival in patients with unresectable or metastatic GC. However, intratumoral molecular heterogeneity, variable molecular target expression, and loss of target expression have limited antibody use and the durability of response. Often immunogenically “cold” and diffusely spread throughout the peritoneum, GC peritoneal carcinomatosis (PC) is a particularly challenging, treatment-refractory entity for current systemic strategies. More adaptable immunotherapeutic approaches, such as oncolytic viruses (OVs) and chimeric antigen receptor (CAR) T cells, have emerged as promising GC and GCPC treatments that circumvent these challenges. In this study, we provide an up-to-date review of the pre-clinical and clinical efficacy of CAR T cell therapy for key primary antigen targets and provide a translational overview of the types, modifications, and mechanisms for OVs used against GC and GCPC. Finally, we present a novel, summary-based discussion on the potential synergistic interplay between OVs and CAR T cells to treat GCPC.

## 1. Introduction

### 1.1. Peritoneal Carcinomatosis

Peritoneal carcinomatosis (PC) is the proliferation and dissemination of malignancy within the peritoneal cavity. Peritoneal dissemination begins with tumor detachment from the primary lesion via spontaneous or metachronous exfoliation. Under Paget’s “Seed and Soil” theory, the fundamental theory of peritoneal dissemination, viable cancer cells travel to favorable sites for metastasis [1]. These cancerous cells seed via trans-mesothelial or trans-lymphatic migration [2]. Following the invasion of the peritoneal layer, the cancer cells proliferate via angiogenesis [3]. Peritoneal stromal tissue—rich in scaffolding, secretory growth factors, and cytokines—is ideal for tumor proliferation. Invasive tumor mechanisms, such as immunomodulation by senescence induction, further allow for metastatic growth. These increase the resistance of PC cells against recognition and clearance by the innate immune system [4]. Intraperitoneal (IP) dissemination is mechanistically aided by gravity, peristaltic movement, and negative intra-abdominal pressure [2]. A poor prognostic indicator in multiple gastrointestinal malignancies, PC leads to over 50–60% of GC-related deaths with a median survival of several months and a 5-year overall survival (OS) rate below 5% [1,2,3].

### 1.2. Current Treatments and Their Inadequacy

Intravenous (IV) systemic chemotherapies have limited efficacy in treating macroscopic GCPC. Several barriers are implicated. Physiologically, poor vascular penetration of the plasma-peritoneal barrier prevents adequate drug delivery into the peritoneal cavity [5,6,7]. Low-oxygenation conditions within the peritoneum contribute to tumor cell hypoxia, which has low apoptotic potential [8]. Moreover, the systemic doses required to achieve sufficient IP effect result in unacceptable systemic toxicity in an already reduced metabolic and excretory capacity secondary to disease burden [9,10]. Lastly, the immunosuppressive tumor microenvironment (TME) that fosters cancer progression contributes to chemotherapy failure and is a significant challenge for achieving peritoneal efficacy even with emerging FDA-approved immunotherapeutic agents for GC. 

Peritoneal-directed regional therapy is another strategy that is studied, particularly among surgical oncologists who view PC as a loco-regional disease state. One notable peritoneal-directed approach combines cytoreductive surgery (CRS) and hyperthermic intraperitoneal chemotherapy (HIPEC). Since the penetration limit of chemotherapeutic agents in HIPEC is approximately 3–5 mm [11,12], the goal of CRS with HIPEC is to eliminate macroscopic tumor deposits by CRS, followed by the removal of microscopic disease via direct peritoneal delivery of heated chemotherapy with hydrophilic and/or ionized high molecular weight drugs. This allows increased concentrations of therapeutic agents to penetrate the plasma-peritoneal border and enhance local tumor cell destruction while limiting systemic toxicity. Compared to other cancer types studied, including appendiceal and colorectal, a durable response to CRS-HIPEC has been difficult to achieve. Unfortunately, the survival benefits of CRS-HIPEC for GCPC are limited to those with microscopic peritoneal disease or low PC index (PCI) scores [13]. 

CRS-HIPEC is challenging for the patient and surgeon being heavily dependent on patient selection and center experience. Adequate cytoreduction is essential, with significant differences in survival based on obtaining a complete CC-0 resection versus CC-1 to CC-3 resection. For example, one study found a 5-year OS rate of 13% for complete cytoreduction compared to 2% with incomplete cytoreduction [14]. The morbidity of CRS and HIPEC is also relatively high, with associated complications ranging from respiratory failure, pleural effusions, pneumonia, anastomotic leaks, intraabdominal abscesses, renal failure, hepatic dysfunction, to bone marrow suppression [15,16]. Although minimally invasive techniques that facilitate multiple HIPEC administrations and decrease the morbidity of CRS are under investigation [17], CRS-HIPEC is not part of the standard-of-care practice for treating GCPC patients. 

More effective therapies are urgently sought to prevent lives lost to GC and GCPC, and a comprehensive strategy is required to overcome the challenges posed by this complex disease. In this study, we provide the most up-to-date review on the pre-clinical and clinical efficacy of CAR T cell therapy for key primary antigen targets in GC and provide a translational overview of the types, modifications, and mechanisms of the OVs used against GC and GCPC. Finally, we present a novel, summary-based discussion on the potential synergistic interplay between OVs and CAR T cells to treat GCPC.

## 2. CAR T Cell Immunotherapy for Peritoneal Carcinomatosis of Gastric Origin

### 2.1. CAR T

Chimeric antigen receptor (CAR) T cell therapy utilizes engineered T cells with tumor-antigen-specific receptors. CARs are fusion proteins that consist of extracellular, hinge, transmembrane, and intracellular signaling domains [18]. The extracellular component is typically an antibody-derived single-chain variable fragment, which serves as the antigen-binding domain, whereas the intracellular component contains T cell signaling domains and additional costimulatory domains [19]. The presence of modified intracellular domains obviates the need for costimulation or major histocompatibility complex (MHC) expression for T cell effector function as antigen binding to the CAR activates signaling. 

Eliminating tumor targets via antigen recognition using CARs provides a significant antitumoral advantage, as the downregulation of MHC expression/loss of MHC-antigen presentation complex is a major cancer immunoevasion mechanism [20]. Later improvements to CAR T cells include the addition of co-stimulatory domains in the second and third generation of CARs, which enhance cytotoxicity, persistence, and expansion of T cells [21]. Fourth-generation CAR T cells, or TRUCKS (T cells redirected for universal cytokine-mediated killing), are additionally equipped with transgenic cytokines such as interleukin-12 (IL-12), which stimulate a potent anti-tumor immune response [22,23]. 

In addition, there are CAR natural killer (NK) cells, which are NK cells engineered to express CARs. Unlike CAR T cells, NK cells have innate, non-specific anti-tumoral cytotoxic capacity, allowing target cell death independent of tumor antigen recognition. These properties further enhance target tumor cell recognition even in immune escape scenarios with tumor antigen downregulation. Also, CAR NK cells can exert cell killing after contact with multiple target cells, though they are short-lived. CAR T cells, in comparison, require stimulation and expansion to kill target cells but can proliferate/persist. Because of their short lifespan, NK cells avoid on-target/off-tumor toxicity seen with CAR T cell persistence, although at the expense of potential longer-term anti-tumor durability [24,25].

First developed in 1989, CAR T cells were clinically evaluated in 2005 in Rotterdam for metastatic renal cell carcinoma and by the National Cancer Institute for metastatic ovarian cancer [26,27]. However, it was not until several years later, when CD19 CAR T cells were tested in refractory follicular lymphoma and advanced leukemia patients, that they demonstrated therapeutic benefits [27,28]. Since then, CAR T cells have delivered promising results in hematologic malignancies, with the first approval of CAR T cell therapy for treating B-cell malignancies in 2017 [29]. Unfortunately, CAR T cells face some barriers in efficacy against solid tumors, but many are currently being investigated in active clinical trials.

### 2.2. Clinical Evaluations of CAR T Cell Targets for GC and GCPC

#### 2.2.1. Claudin 18.2

Claudin 18.2 (CLDN18.2) is a gastric-specific receptor isoform expressed in at least 60% of GC. Though it is highly expressed in normal gastric cells, CLDN18.2 is strictly confined to differentiated epithelial cells in the gastric mucosa [30]. Thus, for GC patients who can undergo total gastrectomy, CLDN18.2-CAR T cells should have limited on-target, off-tumor toxicities. A pre-clinical investigation of CLDN18.2-CAR T cell therapy efficacy in GC cell lines and xenograft murine models has demonstrated potent anti-tumor activities (Table 1) [31]. 

Several clinical studies have also assessed the utility of CLDN18.2-CAR T cells. In a phase I clinical trial for the treatment of CLDN18.2 positive, lymphodepleted gastric and pancreatic cancer, Zhan et al. showed minimal adverse events except for moderate leuko-suppression. These findings suggest that CLDN18.2-CAR T cell therapy has a favorable safety profile [40]. Another CLDN18.2-CAR T cell phase I clinical trial reported similar results, showing only moderate hematologic toxicity without treatment-related deaths or dose-limiting toxicities. Moreover, Qi et al. found in their phase 1 clinical trial that the disease control and overall response rates reached 75.0% and 57.1% in heavily pre-treated GC patients with an OS rate of 81.2% at 6 months, demonstrating promising therapeutic efficacy and acceptable safety despite consistent presence of hematologic toxicity (Table 2) [41].

#### 2.2.2. HER2

HER2 (human epidermal growth factor receptor 2) is a cell surface protein with tyrosine kinase function involved in tumor proliferation, angiogenesis, and metastasis [42]. Overexpression of HER2 is a negative prognostic marker for several solid tumors and is associated with cancer recurrence and treatment resistance [43]. HER2 is overexpressed in 10–30% of all GC [44]. While trastuzumab and other HER2 monoclonal antibodies show nominal efficacy, subsets of HER2-positive tumors demonstrate significant resistance, and adverse effects like cardiotoxicity also limit the extent of HER2 targeting antibodies [45]. Given CAR T cell therapy for hematologic malignancies is generally well tolerated, there is much interest in whether HER2-CAR T cell therapy is an equally efficacious solid tumor treatment modality that can overcome treatment resistance. 

Several murine xenograft models have shown the efficacy of HER2-CAR T cell therapy [33] (Table 1). For example, patient-derived GC cells with low levels of HER2 were minimally affected by HER2-CAR T cells, whereas HER2high GC cells had significantly higher average killing and cytokine release when incubated with HER2-CAR T cells, indicating strong on-target specificity and minimal off-target effect with level-dependent activation. Additionally, CAR-encoding DNA copy numbers on qPCR were detectable beyond 50 days in surviving mice, suggesting the persistence of HER2-CAR T cells [46]. Beyond primary solid tumor efficacy, HER2-CAR T cells have also recently shown potential for the treatment of metastatic solid tumors. Priceman et al. demonstrated enhanced antitumor efficacy of HER2-CAR T cells armed with costimulatory signaling domains in a breast cancer xenograft mouse model with brain metastases. These HER2-CAR T cells not only reduced T cell exhaustion phenotypes but also enhanced proliferation. HER2-CAR T cells had potent antitumor efficacy when delivered intracranially to orthotopic tumors and intraventricularly against multifocal and leptomeningeal metastatic disease [47]. Although clinical efficacy of HER2-CAR T cell therapy has been shown in solid tumors such as rhabdomyosarcoma, pancreatic cancer, and biliary tract cancer [48,49], clinical studies in GC with HER2-CAR T cells are ongoing (Table 2).

#### 2.2.3. Mesothelin

Mesothelin is a cell surface protein typically restricted to mesothelial cells such as those found in the peritoneum, pericardium, pleura, or parts of the reproductive tract. It is significantly upregulated in solid tumors such as triple-negative breast cancer (TNBC), pancreatic cancer, cholangiocarcinoma, mesothelioma, and GC. Although its exact function is unknown, mesothelin is associated with cancer cell adhesion, tumor progression, survival, proliferation, and resistance to apoptosis. Mesothelin-targeting CAR T cells and NK cells show robust anti-tumor efficacy against cancers in human xenograft models, such as TNBC, pancreatic, malignant pleural mesothelioma, ovarian, and GC. In one mesothelin-CAR T cell study, GC cells were eliminated in subcutaneous and IP murine models with considerable NK cell infiltration, while a separate study showed potent cytotoxicity and cytokine secretion [35,50]. Furthermore, adding PH20—a human hyaluronidase that controls tumor progression—enhanced mesothelin-CAR T cell activity against GC (Table 1) [37]. 

However, current clinical trials for solid tumors such as GC, lung cancer, mesothelioma, ovarian, and pancreatic adenocarcinoma using mesothelin-CAR T cells have shown some limitations. While one phase I clinical study for chemotherapy-refractory malignant pleural mesothelioma, pancreatic adenocarcinoma, and ovarian carcinoma showed most patients maintained stable disease after administration with limited toxicity, there was minimal CAR T cell persistence after 28 days [51]. More approaches are needed to enhance anti-tumor efficacy and persistence for mesothelin-CAR T cells. One such method might be in combination with other immunotherapies, such as that seen in a phase I trial of regional mesothelin-CAR T cells with pembrolizumab in patients with malignant pleural disease (Table 2). In the trial with 18 malignant pleural disease patients, stable disease was seen for ≥6 months in 8 patients, and 2 had a complete metabolic response with minimal toxicity and persistence of CAR T cells over 100 days for 39% of patients [52]. Currently, there are no GC-specific clinical trials using mesothelin-CAR T cells, although one ongoing mesothelin-CAR T cell phase I trial (NCT03941626) includes GC in its intended cohort.

#### 2.2.4. NKG2D 

Natural killer group 2D (NKG2D) is an essential activating receptor in NK cells and some T cells, and its ligands are specifically expressed on tumor cells as part of stress-induced tumor immunosurveillance. NKG2D-targeted therapy could have therapeutic applications by engaging NKG2D ligands on tumor cells, potentially inducing cell-mediated cytotoxicity [53]. Moreover, interaction with the ligand triggers immune cell activation, expansion, and pro-inflammatory cytokine reactions, resulting in targeted cell death [54]. Tao et al. showed potent anti-tumor activity of NKG2D-CAR T cells against GC in vitro and in vivo [38]. The study also established that cisplatin upregulated NKG2D expression in GC cells, enhancing NKG2D-CAR T cell-mediated cytotoxicity (Table 1). These findings suggest promising synergy with several avenues of treatment.

#### 2.2.5. PD-L1

Programmed death ligand 1 (PD-L1) is a ligand of the immune checkpoint programmed cell death protein-1 (PD-1) that inhibits cancer cell death. Monoclonal antibodies targeting PD-1 are currently standard-of-care for many tumors but are associated with grade 3–4 adverse events in 7–12% of patients [55]. PD-L1-CAR T cells augment the anti-tumor response beyond a checkpoint blockade and are better tolerated than other monoclonal antibody therapies. 

Some pre-clinical studies suggest PD-L1-CAR T cells can suppress tumor activity without notable toxicity in non-small cell lung carcinoma, GC, and hepatic carcinoma [56,57]. For GC specifically, PD-L1 targeting high-affinity NK cells showed anti-tumor efficacy against 20 human cell lines [58]. Bi-specific Trop2/PD-L1-CAR T cells were also successful in a GC xenograft model (Table 1) [39]. Interestingly, PD-L1 CAR T cells showed a self-amplification phenomenon where they induced PD-L1 expression only by co-culture with cells, as did other effector CAR T cells, resulting in self-propagating PD-L1 targeting activity. Notably, there was potent normal cell cytotoxicity due to bystander PD-L1 induction by PD-L1-CAR T cells [59]. 

Ultimately, more models are needed to determine which cancers PD-L1-CAR T cells can be used against and whether more caution is required in using these for treatment. Some studies suggest high anti-tumor efficacy of PD-L1-CAR T cells without notable toxicity, theoretically surmounting a drawback of antibody therapy with the promise of augmented effect with combination treatment. Others suggest there may be undesired effects. A phase II clinical trial of irradiated PD-L1 CAR-NK cells with pembrolizumab and N-803 against advanced gastric or head/neck cancer (Table 2) and a phase I dual-targeting HER2 and PD-L1 CAR T cell trial against patients with HER2-positive cancer with pleural or peritoneal metastases are ongoing. A separate PD-L1-CAR T cell trial for non-small cell lung cancer was terminated due to an undisclosed adverse event [60].

#### 2.2.6. Carcinoembryonic Antigen

Carcinoembryonic antigen (CEA) is a glycoprotein expressed in various gastrointestinal and stromal tumor types, including GC. Elevated CEA correlates with poorer outcomes in breast and colon cancer [61,62]. CEA is typically expressed only on the luminal face of gastrointestinal cells. Thus, it is not normally accessible to circulating antibodies [63]. Yet, in tumor invasion, CEA is expressed on the entire cell surface and is an accessible epitope for receptor recognition [64]. The immunogenicity of CEA is relatively low; therefore, endogenous T cells do not target CEA [65,66]. In contrast, CAR T cells can overcome this immune tolerance [67]. 

Despite CEA being present in normal cells, a clinical study by Katz et al. demonstrated minimal therapy-related grade 4 or 5 adverse effects when CEA-CAR T cells were used for CEA-positive colorectal liver metastases [68]. In mice, CEA-CAR T cells improved advanced GC survival and limited tumor growth [69]. However, CEA-CAR T cell therapy alone has limited effect against solid tumors and is being investigated with adjunct effectors to support anti-tumor activity. Initial testing with an adjunct recombinant IL-12 with CEA-CAR T cells showed enhanced CAR T cell activation and cytotoxicity in vitro with tumor growth inhibition and CAR T cell proliferation in colorectal, pancreatic, and gastric murine models than CEA CAR T cells alone (Table 1) [62]. Although previous studies provide promising results for improving systemic persistence for CEA-CAR T cells, these are tempered by early on-target off-tumor toxicity concerns. In a phase I clinical trial, some patients experienced acute respiratory toxicity, mandating premature trial closure after the administration of CAR T cell therapy with fludarabine pre-conditioning and infusion of systemic IL-2. CEA expression on lung epithelium coupled with elevated cytokine release were possible factors for transient treatment toxicity [70]. Enabling CEA-CAR T cell persistence while consistently limiting toxicity is challenging.

#### 2.2.7. CD19

CD19 is a biomarker for B-cell development. CD19-targeted therapies are widely successful against hematologic malignancies but are not typically used against solid tumors as CD19 is highly limited to B-cells [71,72]. However, recent pre-clinical studies have successfully used CD19-CAR T cells in solid tumors by presenting CD19 as a target. Though a GC model was not used, CD19-CAR T cells were efficacious against several tumor xenograft mice models when combined with an OV that produced truncated CD19t in infected cells. There was notable induction of local immunity, suggesting that CD19-CAR T cells can be effectively combined with another therapeutic modality [73]. CD19-CAR T cell therapies, however, are still cautiously being considered, as up to 15% of patients can develop malignancies following CAR T cell treatment in hematologic disorders [74]. One case of post-CD19 CAR T cell therapy involved a CAR T cell-treated synchronous follicular lymphoma patient who developed gastric adenocarcinoma mid-treatment. Although complete response was achieved for both conditions with no meaningful impact on quality of life, this is a cautionary example of the clinical drawbacks of using CD19 as a target [75]. While promising, CD19 CAR T cells require more pre-clinical and clinical studies against all tumor types, not just GC. Also, given CAR T cells are limited due to requiring delivery of CD19 as a target in solid tumors first, as CD19 is not a naturally occurring solid tumor target, more data are needed to determine efficacy and safety.

#### 2.2.8. EpCAM

Clinical trials for EpCAM, a transmembrane glycoprotein involved in cell–cell adhesion overexpressed in over 90% of GC are also underway [76]. These are backed by promising results from pre-clinical studies. Zhang et al. recently found that EpCAM-CAR T cells targeted colon cancer cells in an EpCAM-dependent fashion with no systemic toxicity in mice despite eliciting the secretion of cytotoxic cytokines and delaying tumor formation and growth in xenograft models [77]. Another study showed that EpCAM-CAR T cells had potential even for stage IV disease, where intratumoral (IT) injection of these cells into intracerebral lung carcinoma tumors reduced tumor growth and increased murine survival [78]. More specifically for GC, while EpCAM-CAR T cells alone reduced or eliminated tumor burden but were susceptible to relapse, bispecific CAR T cells targeting EpCAM and ICAM-1 significantly prevented relapse and decreased tumor size in gastric, lung, breast, and pancreatic cancer [79]. An ongoing clinical trial is investigating the safety and efficacy of intraperitoneal infusion of EpCAM CAR T cells in GCPC [80]. It is essential to await the results of more pre-clinical and clinical studies of EpCAM CAR T cells before deciding if they are a viable therapeutic candidate. A murine EpCAM CAR T cell study provided counterevidence that these cells may cause lethal toxicity, as cell infusion resulted in dose-dependent cytokine release syndrome, weight loss, and death in tumor-bearing and tumor-free mice [81].

#### 2.2.9. Other Targets

Other potential targets under clinical investigation include CD44, a marker for GC tumor burden and metastasis; CD276, an immune checkpoint molecule whose aberrant expression is associated with tumorigenesis; EGFR, a protein involved in cell signaling pathways overexpressed in 27–64% of gastric tumors; ICAM, a transmembrane glycoprotein involved in inflammatory processes and the T cell-mediated host defense system (Table 1) [34]; MUC1, an oncogene with roles in tumor formation and progression (Table 2); and ROR2, a highly pleiotropic receptor that impacts cell migration and invasiveness (Table 2) [82,83]. Additionally, there has been successful targeting of TAG72, a glycoprotein on the surface of cancer cells, in ovarian and peritoneal ovarian tumors [84]. This approach could be applied to GC and GCPC, as TAG72 is also a GC marker [80].

### 2.3. Challenges Facing CAR T Therapy

In contrast with their hematologic counterparts, solid tumors have a significantly more limited response to CAR T cell therapy. Several factors play into these limitations. Firstly, many solid tumors lack a homogenous, unique antigen as a target. The antigen heterogeneity makes these tumors challenging to recognize, as even though a tumor-associated antigen may be enriched on tumors, they are still expressed at low levels in normal cells [85,86]. This lack of target specificity increases the potential for on-target, off-tumor toxicity that can be fatal without modulating factors added [87,88]. Secondly, solid tumors are also resistant to infiltration by CAR T cells. This is multifactorial from physical barriers (fibroblasts creating dense matrices; atypical vasculature preventing lymphocyte trafficking due to vessel dilation, leakiness, disorganization, tortuosity, irregular flow, and suppression of endothelial adhesion molecules needed for diapedesis) to chemokine and growth factor signaling mismatches or blockades [89,90,91,92,93]. 

Even if CAR T cells recognize and infiltrate solid tumors, the TME is an immunosuppressive milieu, as it is hypoxic, chronically inflamed, nutrient deficient, and acidic [94]. These result in increased inhibitory ligand presentation, such as PD-L1, increased regulatory T cell and M2 tumor-associated macrophage populations, and more inhibitory agents, such as reactive oxygen species [95,96,97]. CAR T cells also suffer from decreased persistence/exhaustion due to chronic antigen exposure, inhibitory factors/ligand interactions, and detrimental proliferation conditions [98]. 

Finally, CAR T cells are associated with toxicities such as cytokine release syndrome, hemophagocytic lymphohistiocytosis/macrophage activation syndrome, and immune effector cell-associated neurotoxicity syndrome. Their effects can not only be severe but are also, in some cases, fatal [99]. New strategies are called for to improve CAR T cell efficacy and ameliorate side effects.

## 3. Oncolytic Virotherapy for Peritoneal Carcinomatosis of Gastric Origin

### 3.1. Oncolytic Virotherapy

The use of viruses to treat cancer began in the late nineteenth century based on observations that cancer patients who contracted viral illnesses sometimes entered brief periods of remission [100]. However, virotherapy research remained relatively stagnant till the mid-twentieth century when the development and refinement of tissue and cell culture allowed for viral propagation ex vivo [101,102]. Virus engineering for immunotherapy began in the early 1990s, and research has since then advanced the efficacy of OVs.

An OV is a naturally occurring or genetically engineered virus that preferentially replicates within and lyses cancer cells [103,104]. By capitalizing on dysregulated signaling pathways in tumor cells to enhance their replication and survival, OVs largely avoid propagating in normal cells. Cancer cells also lack protection mechanisms against viral infection, such as an intact IFN-β signaling pathway, making them more vulnerable to infection [105]. 

There are numerous mechanisms for OV-mediated anti-tumor effects. OVs can inhibit nucleic acid and protein production, limiting cancer cell survival and growth. Viral anti-angiogenic properties induce vascular collapse and contribute to tumor cell death while preventing growth or invasion [106]. Further, OVs can surmount the immunosuppressive TME by mediating the release of innate immunogenic signals, including tumor-associated antigens (TAAs), viral pathogen-associated molecular patterns (PAMPs), and cell-derived damage-associated molecular patterns (DAMPs) [107,108]. Active signaling that promotes type I interferon and cytokine production amplifies the adaptive immune response, recruiting effector immune cells and perpetuating immune memory to provide durable anti-tumor recognition to limit recurrence [109].

There are different OVs currently in clinical trials and practice, and more are under investigation. Furthermore, well-established viruses in oncolytic therapy continue to be refined, such as oncolytic or cancer-specific cytotoxic effect, improved targeting, reduced off-target toxicity, or encoding additional genes. Each virus type in oncolytic therapy has unique benefits and barriers to usage. Thus far, no predominant OV has been approved for GC treatment like T-VEC for melanoma. However, many studies support the role of OVs in GC treatment.

### 3.2. Clinical Evaluations of Oncolytic Virotherapy for GC and GCPC

#### 3.2.1. Herpes Simplex Virus

The herpes simplex virus (HSV) is a neurotropic DNA virus and a member of the alpha-herpes virus subfamily. The first virus to be developed into an oncolytic viral vector and one of the most widely studied, HSV is noted for its ability to replicate quickly in multiple cancer cell types and evade a reactive immune response by the host. Moreover, its large genome allows for easy modification and insertion of multiple transgenes [110]. As a double-stranded DNA virus, HSV is also relatively genetically stable with a polymerase with a low mutation rate compared to other viruses.

In vitro and in vivo studies of G207, a multi-mutated replication-competent HSV type-1, showed that regional viral delivery had tumoricidal effects and prolonged survival in a GC murine model (Table 3) [111]. Likewise, combination therapy involving HSV-1 mutant hrR3 and bevacizumab (an anti-VEGF monoclonal antibody) reduced tumor growth in GC [112]. The viral spread was enhanced by bevacizumab-mediated inhibition of virus-induced angiogenesis, with increased IT dissemination of hrR3. Another study evaluating G207 and HSV1020 found that both viruses reduced tumor burden when administered intraperitoneally––but not intravenously––at higher doses [113]. Against peritoneally disseminated gallbladder cancer, the combination therapy of G207 with 5-fluorouracil prolonged survival in hamsters (Table 3) [114]. Although systemic administration of herpes OVs was less efficacious, these studies suggest local delivery and spread of HSV-based OVs can enhance the treatment of disseminated peritoneal disease or locally aggressive solid tumors.

Beyond exacting purely cytotoxic, lytic mechanisms on tumor cells, HSV-based OVs have been harnessed to enhance diagnostics and have different iterations of modifications enabling the evaluation of treatment effects. One such OV is NV1066, an HSV-1 oncolytic mutant expressing enhanced green fluorescent protein (EGFP). This fluorescent tag allows for laparoscopic visualization in IP tumors, enabling easier detection and localization of the OV. Moreover, the addition of EGFP does not inhibit the anti-tumor properties of the OV. Stanziale et al. showed the cytotoxic effects of NV1066 against GCPC in vitro and in vivo [115].

**Table 3:** Preclinical studies in Oncolytic Viruses for GC and PCGC.

Author (Year)	Oncolytic Virus	In Vitro	In Vivo	Dosing	Results
Zhou et al. (2017) [116]	Ad/TRAIL-E1	Human gastric cancer cell lines (MKN45, MKN28, HGC27, SGC7901), normal human fibroblast (NHFB), normal human gastric epithelial cell line (GES-1)	Xenograft peritoneal GCPC nude mice models (*n* = 15) 5 groups (*n* = 3 per group): PBS, Ad/GFP, Ad/GFP-E1, Ad/gTRAIL, ad/TRAIL-E1 at 4 d after tumor inoculation	In vitro: MOI 30–3000 In vivo: 3 × 10^10^ PFU IP every 4 days × 3	Induction of TRAIL-mediated apoptosis in GC lines only (*p* < 0.01) Inhibition of peritoneal metastases, lower tumor weights with Ad/TRAIL-E1 (*p* < 0.05) Prolonged survival (83 days) compared to control (PBS—46 days) or Ad/CMV-GFP treatment (55 days) (*p* < 0.01)
Haley et al. (2009) [117]	EV1	Human gastric cancer cell lines (AGS, Hs746T, NCI-N87, MKN45), human ovarian cancer cell line (DOV13)	Xenograft peritoneal GCPC NSG mice models (*n* = 40) 5 groups (*n* = 8 per group): weight control (no injections), PBS, 3 different EV1 doses 5 d after tumor inoculation	In vitro: Varied MOIs In vivo: 1 × 10^3^ TCID_50_, 1 × 10^5^ TCID_50_, or 1 × 10^7^ TCID_50_ IP	Therapeutic dose-dependent tumor regression by day 35 after treatment (*p* < 0.01) Viable bioluminescent model of GCPC for non-invasive peritoneal tumor burden monitoring
Jun et al. (2014) [118]	GLV-1 h153	Human gastric cancer cell lines (AGS, OCUM-2MD3, MKN74, TMK-1)	Xenograft subcutaneous GC nude mice model (*n* = 10) 2 groups (*n* = 5 per group): PBS vs. GLV-1 10 d after tumor inoculation	In vitro: MOI 0.01–1 In vivo: 2 × 10^6^ PFU IT	>70% cytotoxicity in vitro in all GC lines Xenograft regression by day 15 (*p* < 0.01) Readily imaged tumor infection on SPECT
Bennett et al. (2000) [111]	G207	Human gastric cancer cell lines (AGS, MKN1, MKN74, MKN45P, OCUM-2MD3)	Xenograft peritoneal GCPC nude mice model (*n* = 50) 5 groups (*n* = 10 per group): PBS, low dose G207 3 h or 3 d after tumor inoculation, high dose G207 3 h or 3 d after tumor inoculation	In vitro: Varied MOIs In vivo: 5 × 10^6^ PFU or 5 × 10^7^ PFU IT	>70% cytotoxicity in vitro in all GC lines at MOI 0.1 by 96 h G207 with tumor burden reduction at all timepoints at 5 × 10^7^ PFU and at 3 h with 5 × 10^6^ PFU (*p* < 0.01) with improved survival at all timepoints after virus injection (*p* < 0.01)
Nakano et al. (2005) [114]	G207	N/A	Xenograft peritoneal GCPC NSG mice model (*n* = 25) 3 groups: control (*n* = 10), G207 (*n* = 9), G207 + 5FU (*n* = 6) at 10 d after tumor inoculation	In vivo: 1 × 10^7^ PFU IP	Improved survival of mice treated with G207 and G207 + 5FU compared to control (*p* < 0.01), although no significant differences between G207 versus and 5FU monotherapy (*p* = 0.08)
Sugawara et al. (2020) [119]	G47Δ	Human gastric cancer cell lines (MKN1, MKN45, MKN74, NUGC4, OCUM1, Kato III, HSC60, HSC39, 44As3)	Xenograft subcutaneous, orthotopic, and peritoneal GC and GCPC nude mice models Subcutaneous (*n* = 7 per group): mock, low dose, high dose G47Δ at day 0, 3 Intratumoral (*n* = 7 per group): mock, G47Δ at day 8, 11 GCPC: mock (*n* = 10), G47Δ at two doses (*n* = 10 per dose) on days 3, 5, and 7	In vitro: MOI 0.01 to 1 In vivo: - Subcutaneous: low dose 2 × 10^5^ or high dose 1 × 10^6^ PFU IT - Orthotopic: 1 × 10^6^ PFU IT - GCPC: 1 × 10^6^ or 5 × 10^6^ PFU IP	Inhibition of orthotopic tumor growth with G47Δ even at low dose (*p* = 0.04 at day 27) Improved survival of G47Δ treated orthotopic GC mice compared to mock-infected mice (68 days vs. 55 days, *p* < 0.01) Significant prolongation of survival of GCPC mice with IP G47Δ treatment as low as 1 × 10^6^ PFU, with all mock treatment mice dying at day 30 with 3 and 6 mice surviving at day 50 at 1 × 10^6^ PFU and 5 × 10^6^ PFU treatment regimens, respectively (*p* < 0.01)
Deguchi et al. (2012) [112]	Oncolytic Herpes Virus	Human pancreatic cancer (Capan1, MiaPaCa2), hepatic cancer (Hep3B, PLC/PRF/5), gastric cancer (AZ521, MKN45), colon cancer (WiDr), ovarian cancer (SKOV3) cell lines	Xenograft subcutaneous GC nude mice models (*n* = 24) 4 groups (*n* = 6 per group): PBS, hrR3, Bevacizumab, hrR3 + Bevacizumab twice weekly for two weeks	In vitro: MOI 0.01 to 10 In vivo: hrR3 1 × 10^7^ PFU IT and Bevacizumab 100 mg/mouse intracisternally	Bevacizumab did not impact viral cytotoxicity even at 10 mg/mL Combination bevacizumab and hrR3 inhibited tumor growth compared to control or bevacizumab alone (*p* < 0.003) and hrR3 alone (*p* < 0.001) by day 49 Combination treatment reduced angiogenesis compared to control (*p* < 0.07) and hrR3 alone (*p* < 0.001) but hrR3 alone had higher angiogenesis than control (*p* < 0.001)
Zeng et al. (2011) [120]	Vesicular Stomatitis Virus	Human gastric cancer cell (MKN28)	N/A	In vitro: N/A—utilized viral protein only	Vesicular stomatitis virus matrix protein induced cancer cell apoptosis, likely secondary to triggering rapid intracellular ROS accumulation
Sui et al. (2017) [121]	Newcastle Disease Virus	Human gastric cancer cell lines (BGC823, SGC7901, MKN28)	Xenograft subcutaneous GC nude mice models (*n* = 15) 3 groups (*n* = 5 per group): mock, pre-tumor inoculation GC cell NDV-D90 infection, post-tumor inoculation GC cell NDV-D90 infection	In vitro: MOI 0.001 to 10 In vivo: Viral dose not specified, IT	Dose-dependent NDV-D90 cytotoxicity NDV-D90 with higher replication and anti-tumor effect in low differentiated, highly proliferative GC (*p* < 0.05 for SGC7901 and BGC823 vs. MKN28) NDV-D90 significantly impairs GC vascularization, demonstrated objectively by VEGF-A and MMP-2 levels (*p* < 0.05)
Song et al. (2010) [122]	Newcastle Disease Virus	Human gastric cancer cell lines (AGS, MKN74)	Xenograft peritoneal GCPC NSG mice models Toxicity: 3 NDV(F3aa)-GFP dosage groups GCPC (*n* = 20): PBS (*n* = 5), single NDV(F3aa)-GFP dose at day 1 (*n* = 7), 3× NDV(F3aa)-GFP doses at days 1, 4, and 7 (*n* = 8)	In vitro: MOI 0.01 to 1 In vivo: Toxicity—2 × 10^6^, or 5 × 10^6^, or 1 × 10^7^ PFU IP GCPC—5 × 10^6^ PFU IP	>30% cell death by day 7 at MOI 0.01 for MK74 treated with NDV(F3aa)-GFP but no cytotoxicity, minimal infection for AGS cells No toxicity noted at all doses for NDV(F3aa)-GFP in vivo Significant reduction in peritoneal tumor burden for NDV(F3aa)-GFP treated mice compared to control (7.22 ± 1.59 g control; 1.46 ± 2.06 g single-treatment; 1.36 ± 2.01 g multiple-treatment group; *p* = 0.005)
Bennett et al. (2002) [113]	Herpes Simplex Virus	Human gastric cancer cell lines (MKN45, OCUM-2MD3, MKN74)	Xenograft peritoneal GCPC nude mice models Regional treatment (*n* = 190): OCUM-2MD3 and MKN45-P cells, 5 groups: 4 different dosages of G207 or NV1020, PBS Systemic (*n* = 50): OCUM-2MD3 cells, 5 groups—single dose G207 or NV1020 (*n* = 9 per virus), 3× dose G207 or NV1020 every other day (*n* = 10 per virus), control (*n* = 10) Survival (*n* = 40): OCUM-2MD3 cells, 5 groups (*n* = 8 per group): control, G207 low, G207 medium, NV1020 low, NV1020 medium dose	In vitro: MOI 0.01 to 1 in vivo: 2.5 × 10^6^, or 5 × 10^5^, or 5 × 10^6^, or 5 × 10^7^ PFU IP	NV1020 more cytotoxic at all MOIs than G207 (*p* ≤ 0.05) for all GC lines and with higher viral proliferation in all cell lines (*p* < 0.05) IP Treatment with G207 and NV1020 ≥ 2.5 × 10^6^ PFU reduced GCPC burden (*p* ≤ 0.05) for MKN45-P and OCUM-2MD3 models IV treatment of OCUM-2MD3 GCPC did not cause any reduction in tumor burden regardless of dosage or number of doses IP treatment of low or medium dose NV1020 improved median survival (49 and 48 days vs. 32 days, *p* < 0.01 and *p* < 0.02, respectively), but no G207 dose showed significant survival advantage compared to controls (low dose 36 days, medium dose 39 days, *p* = NS), and NV1020 dose effects were significantly different than G207 dose effects (*p* < 0.03 all)
Matsumura et al. (2021) [123]	Herpes Simplex Virus	Human gastric cancer cell lines (MKN1, MKN28, MKN73)	N/A	Ex vivo: MOI 0.01, 0.1	Oncolytic HSV expressing SOCS-3 (T-SOCS3), an HSV with SOCS3 enhanced replication and the killing effect against gastric cancer cell lines compared to second-generation oncolytic HSV T-01
Stanziale et al. (2004) [115]	Herpes Simplex Virus	Human gastric cancer cell lines (OCUM-2MD3)	Xenograft peritoneal GCPC nude mice models (*n* = 40) 5 groups (*n* = 8 per group): single low dose at day; multiple low doses at day 1, 2, and 3; single high dose at day 1; multiple high doses at day 1, 2, and 3, untreated	In vitro: MOI 0.01 to 1 In vivo: 1.5 × 10^6^, 1.5 × 10^7^, multiple doses 1.5 × 10^6^, multiple doses 1.5 × 10^7^ PFU IP	Dose and time-dependent cytotoxicity of OCUM GC cells, unaffected by EGFP construct Peritoneal weights significantly reduced in all treatment groups except the single low-dose group (*p* = 0.22) Significant differences based on dose amounts, with high dose and a greater decrease in tumor burden compared to single and multiple low dose treatments (*p* = 0.04 and 0.01, respectively)
Tsuji et al. (2013) [124]	Herpes Simplex Virus	Human gastric cancer cell lines (AZ521, MKN1, MKN28, MKN45, MKN74, TMK-1)	Xenograft subcutaneous GC nude mice models (*n* = 24 3 groups (*n* = 8 per group): PBS, T-01, or T-TSP-1 at day 5–7 after tumor implantation	In vitro: MOI 0.01 and 0.1 In vivo: 1 × 10^7^ PFU IT	T-01 had minimal tumor growth compared to PBS control group (7× tumor growth, *p* < 0.01), but T-TSP-1 had significant tumor growth inhibition even compared to T-01 (*p* < 0.05) Enhanced cytotoxicity of T-TSP-1 compared to T-01 in vitro for some GC lines, possible from signal transduction of α3β1 integrin
Yano et al. (2013) [125]	Adenovirus	Human gastric cancer cell lines (MKN45, MKN7)	Xenograft subcutaneous GC nude mice tumor models (*n* = 15) 3 groups (*n* = 5 per group, bilateral tumors): OBP-301, cisplatin, radiation, control every 3 days for 3–5 treatments	In vitro: varied MOIs In vivo: 1 × 10^8^ PFU IT	Adenovirus OBP-301 significantly decreased the percentage of CD133^+^ stem-like cells compared to cisplatin or radiation by day 3 after treatment (*p* < 0.05) w/corresponding suppression of CD133 mRNA at 24 h (*p* < 0.05) and reduction in CD33 expression OBP-301 significantly alters quiescent cancer stem-like cell states, mobilizing them to have less percent in G_0_–G_1_ phase and increased proportion in S-phase, with the killing of cancer-like stem-cells in S-phase OBP-301 mobilizes infected quiescent CD133^+^ cells in tumor spheres, eradicating dormant cells not reached by other treatments
Ishikawa et al. (2020) [126]	Adenovirus	Human gastric cancer cell lines (GCIY, Kato III)	Xenograft peritoneal GCPC nude mice models (*n* = 12) 2 groups (*n* = 6 per group): OBP-401 at day 17 after tumor inoculation, PBS	In vitro: MOIs of 0, 1, 2, 10, 25, 50, or 100 In vivo: 1 × 10^5^ PFU IP	Significant antitumor effects paclitaxel and OBP-401 against GCPC, but greatest effect with combination treatment OBP-401 with paclitaxel with decreased peritoneal tumor burden by day 28 and ascites by day 35 compared to PTX or OBP-401 monotherapies (*p* < 0.05 for both, respectively) OBP-401 able to selectively localize and replicate in peritoneal metastatic nodules with reliable fluorescence-based imaging
Xu et al. (2014) [127]	Adenovirus	Human gastric (NCI-N87, MGC80-3, AGS), liver (Huh-7, SMMC-7721), cervical (HeLa), colon (SW480, HCT116), and pancreatic (BxPC3) cancer cell lines, normal gastric epithelial cell line (GES-1)	Xenograft subcutaneous GC nude mice models (*n* = 30) 5 groups (*n* = 6 per group): saline, Ad.vector, Ad.AChE, ZD55, ZD55-AChE	In vitro: MOIs 1, 10, 25, 50, 100, 200 In vivo: 1 × 10^9^ PFU IT per virus	ZD55-AChE suppressed in vitro and in vivo cell growth in several GC cell lines without toxicity shown in normal gastric epithelial or primary fibroblast cells and more so than replication-deficient adenovirus Ad.AChE Low MOIs of ZD55-AChE also cytotoxic for some pancreatic, colon, and liver cancer cell lines Mechanism of ZD55-AChE cell death partly by induction of mitochondria-based apoptosis
Lv et al. (2019) [36]	Measles Virus	Human gastric cancer cell lines (BGC-823, SGC7901)	Xenograft subcutaneous GC nude mouse model (*n* = 20) 2 groups (*n* = 10 per group): control mock injection, rMV-Hu191 treatment at days 7, 8, 9, 11, 13, and 15	In vitro: MOI 0.1, 0.5, 1, 5, 10 in vivo: 1.4 × 10^7^ TCID_50_ per treatment day	rMV-Hu191 causes dose and time-dependent cytotoxicity for both GC lines tested Induction of caspase-dependent apoptosis both in in vitro and in vivo after infection of virus (decreased caspase 3 activation, *p* < 0.01, and decreased PARP activation *p* < 0.05) Lipid raft integrity required for rMV-Hu191 viral entry and for viral-induced apoptosis 1.76-fold increase in median survival in GC subcutaneous nude mice models after treatment with rMV-Hu191 (*p* < 0.01)
Lv et al. (2021) [128]	Measles Virus	Human gastric cancer cell lines (BGC-823, SGC-7901, GCSR1)	Xenograft subcutaneous GC nude mice model (*n* = 40) 4 groups (*n* = 10 per group): mock, rMV-Hu191 (days 5, 6, 7, 9, 11, and 13 after tumor inoculation), DDP (days 7, 14, and 21), rMV-Hu191 + DDP	In vitro: MOI 0.1, 1 In vivo: 1.4 × 10^7^ TCID_50_ rMV-Hu191; 10 mg/kg DDP per treatment	Combination of rMV-Hu191 and DDP-based chemotherapy treatment had synergistic cytotoxicity in drug-resistant and drug-nonresistant GC cell lines Combination therapy with greater survival than rMV-Hu 191 (median survival 23 days) or DDP (median survival 17 days) alone DDP dose not interfere with viral replication
Kawaguchi et al. (2010) [129]	Reovirus	Human gastric cancer cell lines (MKN45P, NUGC4, MKN7, Kato III), normal epithelial cell line (NIH3T3)	Xenograft intraperitoneal GCPC nude mice model (*n* = 20) 2 groups (*n* = 10 per group): PBS, reovirus	In vitro: MOI 10 In vivo: 1 × 10^8^ PFU, IP	All GC cell lines with significant cytotoxicity within 1 week of reovirus infection whereas normal cells resistant to infection Increased Ras activation after reovirus infection in GC cell lines IP reovirus-treated mice with significantly fewer ascites than non-treated mice (0.14 mL vs. 3.86 mL, *p* < 0.05) Mean peritoneal tumors less in the virus group and control (11.9 vs. 65.2 nodules, *p* < 0.05)
Cho et al. (2010) [130]	Reovirus	Human gastric cancer cell lines (SNU216, SNU668), mouse fibroblast cells (L929)	N/A	In vitro: MOI 1, 10	Apoptosis triggered after reovirus infection in known TRAIL-resistant GC cells Reovirus infection downregulated Ras, ERK, but not p38 MAPK activation

GC: gastric cancer; GCPC: gastric cancer peritoneal carcinomatosis; N/A: not applicable; MOI: multiplicity of infection; PFU: plaque-forming units; TCID_50_: median tissue culture infectious dose 50%; IP: intraperitoneal; IT: intratumoral; IV: intravenous.

A third-generation oncolytic HSV type 1, G47∆, was effective when administered intratumorally and intraperitoneally in advanced GC models, including peritoneal dissemination of scirrhous GC [119]. Notably, the IT injections of G47∆ decreased M2 macrophages, which secrete anti-inflammatory factors, yet conversely increased their pro-inflammatory counterparts, M1 macrophages and NK cells (Table 3). Thus, G47∆ could be a promising therapeutic agent, as it engages and enhances tumor immune response in addition to its oncolytic properties, supplementing the overall anti-tumor effect.

Other HSV modifications include the addition of transgenes to enhance an oncolytic effect, with thrombospondin-1 (TSP-1) and the suppressor of cytokine signaling 3 (SOCS3) as notable examples. TSP-1 is a protein that suppresses tumor growth through various mechanisms, including anti-angiogenesis. Armed with TSP-1, an oncolytic HSV displayed an enhanced antitumor effect against GC models [124]. Likewise, oncolytic HSVs expressing SOCS3 also improved the oncolytic effect, as replication and cytolysis were enhanced in human GC cell lines (Table 3) [123].

#### 3.2.2. Adenovirus

Adenoviruses are large, double-stranded, non-enveloped viruses in an icosahedral capsid [131]. In addition to high genetic stability and low pathogenicity, adenoviruses are easy to produce, making them logistically favorable therapeutic agents [132]. While adenoviruses as viral vectors for gene therapy failed previously secondary to limited delivery, [133] their value as a self-propagating OV was recognized, and the first modified adenovirus virotherapy H101 was approved in China in 2005 for combined chemotherapy and OV treatment of head and neck cancer [134]. Since then, these remain one of the most frequently studied OV, and multiple approaches modifying adenovirus capsids, enhancing tropism and selectivity, small viral gene deletions, tumor-specific promoter insertions, tumor-associated antigen transgene additions, and other immunostimulatory transgenes, have enhanced antitumor potency [135].

Ad/TRAIL-E1 is one of the many oncolytic adenoviruses currently under investigation. It utilizes a tumor-specific promoter (TSP) to control a transgene encoding tumor necrosis factor-related apoptosis-inducing ligand (TRAIL) and the viral E1A gene, which play a role in tumor necrosis and p53-dependent apoptosis, respectively. Notably, TRAIL avoids toxicity in normal cells while specifically inducing cancer cell apoptosis [116]. The E1A gene allows a second mechanism to overcome TRAIL resistance by primary cancers that are sometimes observed [136]. In the study by Zhou et al., Ad/TRAIL-E1 OV significantly increased the survival of mice with advanced GC and inhibited peritoneal metastasis without virus-induced toxicity (Table 3) [116].

OBP-401 is another oncolytic adenovirus with efficacy in GC. The attenuated adenovirus controls viral replication based on the human reverse transcriptase promoter while encoding GFP to visualize viable cancer cells. It works synergistically when administered intraperitoneally with paclitaxel to suppress human GC viability. Paclitaxel enhanced viral penetration into peritoneally disseminated nodules, demonstrated by GFP-positive spots. Combination therapy also inhibited PC growth and reduced malignant ascites burden, suggesting potential symptomatic clinical benefit for PC patients (Table 3) [126]. Even the parental virus OV OBP-301 was shown to mobilize a problematic subgroup of GC cells, the quiescent GC stem cells, resistant to chemoradiation in their dormant status. Via the induction of the cells into the S/G2/M phases, OBP-301 caused the loss of cancer stem-like properties (Table 3). Thus, cells became chemo-sensitive and were eliminated [125]. Ultimately, both OBP-301 and -401 have unique utility, with the former allowing for treatment sensitization of an otherwise resistant subgroup of GC cells that could cause cancer persistence and the latter enabling non-invasive monitoring of treatment efficacy.

Finally, ZD55-AChE is an adenovirus-based OV used in GC models. The therapeutic efficacy was shown where higher AChE—a terminator of acetylcholine signaling transmissions—levels were associated with longer survival in GC patients. Thus, using their oncolytic adenoviral vector ZD55-AChE to overexpress AChE, the authors found ZD55-AChE induced apoptosis in GC cells at low doses and inhibited GC stem cell growth and tumor progression in vivo. The replication-deficient adenoviral vector Ad.AChE was less effective than the oncolytic vector ZD55-AChE, requiring a higher multiplicity of infection for a similar effect, suggesting that the OV form may be more practical (Table 3) [127].

#### 3.2.3. Newcastle Disease Virus

Newcastle Disease Virus (NDV) is an avian RNA virus from the Paramyxoviridae family. Due to the ease of modification, minimum recombination frequency, and rapid replication cycle of all paramyxoviruses, NDV is an appealing platform for developing OV therapies [137,138].

NDV-D90 was shown to increase apoptosis and reduce cell growth in GC cell lines [121]. This NDV suppressed ERK1/2 and Akt signaling, increased p38 signaling, and reduced vascularization of gastric tumors. Orthotopic tumors from cells pre-treated with NDV-D90 also showed impaired tumor development after implantation (Table 3). While the exact mechanisms of all changes were not fully elucidated, the potential anti-GC effects of NDV-D90 were demonstrated.

NDV(F3aa)-GFP is a mutant NDV virus with a modified F cleavage site and inserted EGFP (Table 3). Song et al. administered IP NDV(F3aa)-GFP in a murine GCPC model and conducted in vitro studies using human GC cells. They demonstrated dose-related killing of GCPC cells in vitro, and over half the mice had no disease following treatment. These positive findings provide a further basis for the clinical trials of NDV for GCPC [122].

#### 3.2.4. Vaccinia Virus

The vaccinia virus is an enveloped DNA virus with a large genome and can target the cell cycle, harness apoptotic pathways, and, like all other viruses, induce an immune response [139]. Most well-known for its role in eradicating smallpox, it has found a broadening avenue in oncolytic virotherapy.

GLV-1 h153 vaccinia virus expressing the human sodium iodide symporter (hNIS) for deep-tissue imaging showed effective regression of gastric tumors [118]. Of five GC cell lines, GLV-1 h153 achieved over 90% cytotoxicity in three and over 70% in two at MOI 1 by 9 days while effectively treating xenografts after two weeks of treatment (Table 3). GL-ONC1 was well tolerated following IP administration in a phase 1 study of advanced-stage GC patients. However, efficient infection, viral replication, and tumor lysis were limited to the first treatment cycle (Table 4) [140]. Despite these findings, vaccinia-based OVs in other tumor models continue to show improved replication and efficacy, and newer studies in a GC model may reflect these newly improved, modified vaccinia OVs.

Another vaccinia-based OV, CF33, has also shown much clinical promise. CF33 is a chimeric virus derived from different poxviruses encompassing multiple strains of vaccinia virus, with enhanced potency and safety compared to any of the parental viruses [141]. CF33-derived viruses such as those expressing hNIS (CF33-hNIS) or hNIS with an anti-PD-L1 single chain variable fragment (CF33-hNIS-antiPDL1) have shown antitumoral effects in breast, colon, lung, and pancreatic cancer models [142,143,144]. Both viruses are currently in phase I clinical trials for unresectable solid tumors and TNBC. 

Recently, we demonstrated the significant antitumor activity of CF33 and its derivatives in GC models. CF33, CF33-hNIS-∆, and CF33-hNIS-antiPDL1 exhibited significant dose-dependent infection, replication, and cytotoxicity against multiple human-derived GC cell lines in vitro. In a GCPC xenograft mouse model, IP delivery of CF33-hNIS-antiPDL1 significantly reduced peritoneal metastases, prolonged survival, and largely prevented malignant ascites development. Moreover, the presence of CF33-hNIS-antiPDL1 was not detectable in mouse organs 2 weeks after treatment, suggesting that the virus has significant potential to treat GCPC and its sequelae, and it is safe with minimal to no off-target toxicity [145]. While there are no ongoing trials, this study provides the preclinical basis for potential clinical trials of CF33 in GCPC patients.

#### 3.2.5. Measles Virus

The measles virus (MV) is a negative-stranded RNA paramyxovirus with a genome of approximately 16 kb. MV preferentially replicates in malignant cells, lacks genotoxicity, and has an excellent safety profile, though it also faces antiviral immunity challenges [146].

rMV-Hu191 is a recombinant Chinese Hu191 MV. It induced cytopathogenicity and inhibited tumor growth via caspase-dependent apoptosis in GC models, whereas survival was significantly prolonged in tumor-bearing mice [36]. Later, rMV-Hu191 was also effectively implemented in combination therapy. In conjunction with DDP-based chemotherapy, rMV-Hu191 synergistically induced cytotoxicity in drug-resistant and -nonresistant GC cell lines (Table 3) [128].

Bach et al. demonstrated that MV-141.7 and MV-AC133, which are MVs retargeted to CD133, could infect and lyse CD133+ tumor cells [147]. While the study dealt with hepatocellular carcinoma growing subcutaneously and in the peritoneal cavity, CD133 is also a stem cell marker for recurrence and metastasis in GC. Because of this shared marker, MV-141.7 and MV-AC133 could successfully target CD133+ GC cells in advanced GC and GCPC.

#### 3.2.6. Reovirus

Reovirus, or respiratory enteric orphan virus, is a non-enveloped, double-stranded RNA virus protected by two concentric icosahedral capsids [148]. Naturally oncolytic, reovirus remains cytotoxic to tumors despite the presence of neutralizing antibodies [149]. Further, it can override tumor immune evasion and establish antitumor immunity against subsequent cancers [150]. 

Kawaguchi et al. observed the cytopathic effect of reovirus in mouse GC models with peritoneal metastasis [129]. The mean volume of ascites and total number and weight of peritoneal tumors also decreased in reovirus-treated mice (Table 3). Meanwhile, Cho et al. showed that reovirus downregulates the activation of Akt, a protein kinase that drives oncogenesis, and signaling molecules that promote cell proliferation and survival, including Ras and ERK (Table 3) [130]. Reovirus induced the apoptosis of TRAIL-resistant GC cells via these mechanisms.

Reovirus also demonstrated success when combined with trastuzumab against TRAIL-nonresistant GC cells. In vitro and in vivo studies showed that reovirus induces amplification of TRAIL on cancer cells. Also, it is speculated that reovirus augments the cytotoxic activity of trastuzumab [151]. Further research into reovirus is necessary, as there are mixed results regarding its efficacy in clinical trials for multiple tumor types, and there is concern that the seroprevalence of neutralizing antibodies (present in up to 50–100% of the population) may limit full efficacy [152].

#### 3.2.7. Vesicular Stomatitis Virus

Vesicular stomatitis virus (VSV) is an enveloped, negative-stranded RNA virus in the Rhabdoviridae family. VSV targets cells with defective antiviral interferon signaling pathways, which aids in tumor specificity. However, high dosages of VSV are associated with off-target toxicities [153]. The VSV matrix protein (MP) plays a core role in its antitumor effects. As illustrated in a study of GC, MP expression can promote the rapid buildup of reactive oxygen species (ROS), contributing to tumor cell apoptosis [120].

VSV replicates rapidly, notably enhanced in hypoxic environments, and leads to cytopathic and apoptotic effects [154]. Beyond tumor toxicity and induction of apoptosis, VSV also limits ascites burden, as shown in a GCPC model [155]. The hypoxic environment of malignant ascites promotes increased glycolysis and glutamine metabolism, both of which are necessary for efficient viral replication [154]. Thus, VSV appears well suited for replication and anti-tumor effects in GC, although its off-target toxicities currently limit its ability to be safely used.

#### 3.2.8. Echovirus

The enteric cytopathic human orphan virus (echovirus) is an enterovirus that affects the gastrointestinal tract. The low-pathogenic strain Echovirus 1 (EV1) was shown to interact with α2β1, an integrin required for lytic EV1 infection, and was involved in the peritoneal dissemination of GC. Haley et al. noted multiple GC cell lines amply express α2β1, suggesting increased susceptibility to EV1 [117]. Escalating doses of EV1 showed a therapeutic response in mouse models for gastric PC.

### 3.3. Challenges Facing Oncolytic Virotherapy

While oncolytic virotherapy has promise, numerous challenges, which include antiviral immune response, tumor barriers inhibiting viral penetration and propagation, and the lack of reliable, predictive, specific, and therapeutic biomarkers, limit its efficacy as a monotherapy [99,156]. Moreover, tumor heterogeneity demands a tailored approach involving the appropriate virus and delivery method. Logistically, delivery is challenging to OV therapy, as antiviral immunity hinders systemic delivery, and IT administration is costly and technically difficult [157]. Consequently, several studies using OVs in combination approaches have increased the duration and stability of anti-tumor effects.

## 4. Combination of CAR T and Oncolytic Virus Therapy for Peritoneal Carcinomatosis of Gastric Origin

### 4.1. Rationale for Combination CAR T and Oncolytic Virus Therapy

**Figure 1:**
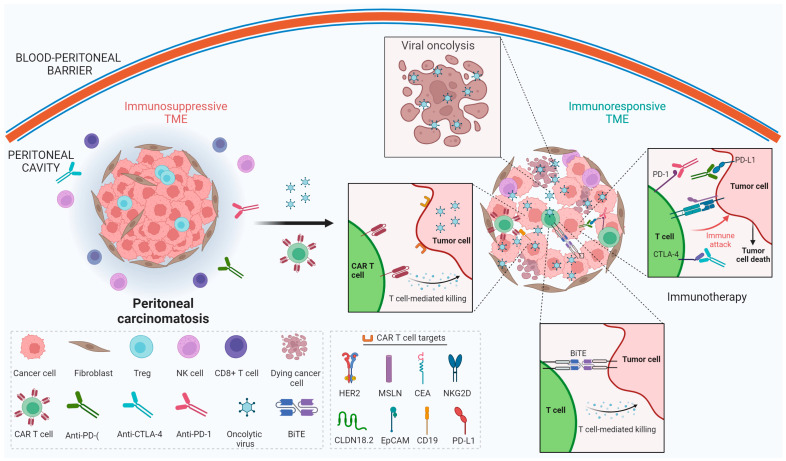
Schematic showing different mechanisms by which oncolytic viruses and CAR T cells could turn an immunosuppressive tumor microenvironment into an immunoresponsive tumor microenvironment in the context of peritoneal carcinomatosis. This image was created using BioRender.

CAR T cell therapy and oncolytic virotherapy remain limited as single-agent therapies against solid tumors, but synergistic efficacy could be expected when combined (Figure 1).

OVs can augment CAR T cell therapy via several mechanisms. One is via type I IFN production by immune cells, which boosts CAR T cell infiltration, stimulation, and proliferation [158]. Type I IFNs increase cytokine production to further propagate an immune response, improve cytolytic function, and promote the differentiation of effector T cells to memory cells, reducing the risk of tumor recurrence [159,160]. Beyond improving efficacy, persistence, and later immune recognition from CAR T cells, oncolytic adenoviruses also destroy cancer stem cells, blunting tumor relapse [161]. Similarly, an oncolytic HSV-TRAIL suppressed tumor progression and cured 40% of treated mice when used with a TGF-β inhibitor, which plays a crucial role in preserving the stemness of glioblastoma stem cells [162]. Thus, OVs can target tumor cell populations that are otherwise difficult to eliminate with chemotherapy or T cell therapy alone, further reducing disease recurrence risk.

A second mechanism that allows for OVs to work favorably in combination therapy is via their potent immunogenic properties—reversing the immunosuppressive TME to convert “cold” tumors into “hot” tumors. Damage-associated molecular patterns (DAMPs) are linked with cancer cell death, and OVs could induce the release of DAMPs along with various immunostimulatory molecules [163,164]. Stress to the endoplasmic reticulum by OVs releases ROS, with consequent increases in protein kinase RNA-like endoplasmic reticulum kinase (PERK)-dependent immunogenic cell death and mitochondrial apoptosis [165,166]. Moreover, OVs can be genetically modified to secrete cytokines, which help create an immunologically “hot” TME. These signals either directly or indirectly recruit immune cells like CAR T cells, improving their propagation and targeting of tumor cells via molecular signaling. 

OVs enhance the tumor selectivity of CAR T cells through the delivery of tumor-selective surface antigens. This is particularly useful for solid tumors to overcome tumor antigen heterogeneity if an amenable antigen exists. These delivered antigens may be targeted by bispecific T cell engagers (BiTEs). A BiTE is a bispecific antibody construct engineered to have two binding domains from peptide-linked single-chain variable fragment regions from antibodies. One domain binds tumor antigens and the other binds the invariant T cell surface target CD3 to increase targeting and engagement of T cells [167,168]. BiTEs that recognize these antigens can interact on the cell surface and redirect adaptively transferred T cells to kill cancer cells and regulate inflammatory responses [169]. Through these mechanisms, among others, BiTEs could strengthen the potency of the combination of CAR T cells with OVs, as demonstrated by oncolytic vaccinia and adenovirus studies [170,171]. OVs can also be modified to deliver therapeutic transgenes to the TME, further enhancing CAR T cell effector functions. Alternatively, recombinant adenoviruses are engineered to express CD19 tags, which mark antigenically deviant tumors for recognition by CD19 CAR T cells. The oncolytic tagging system reduced tumor size in vivo and significantly prolonged survival in murine models [172]. Similarly, Park et al. engineered an oncolytic vaccinia virus to express a nonsignaling, truncated CD19 protein on the tumor cell surface. This promoted self-propagating CD19-CAR T cell tumor targeting and enhanced CD19-CAR T cell proliferation, cytokine production, and function through viral replication and lysis [73].

A pitfall of oncolytic virotherapy is the high dosage required to evade antibodies and other immune responses. The use of CAR T cells as OV carriers––shielding OVs as they travel to target tumor cells from hostile immune components––could solve this problem. Mouse and human CAR T cells loaded with low doses of OV showed no impairment of receptor expression or function. Following the successful deposit of OVs, which did not impact CAR T cell and tumor-cell interaction, tumoricidal activity was enhanced [173].

### 4.2. Clinical Evaluations of Combination of CAR T Cells and Oncolytic Virus Therapy in Solid Tumors

Though combination therapy has not been tried against GC or GCPC, several studies targeting solid tumors have been successful. As mentioned above, Park et al. engineered an oncolytic chimeric orthopoxvirus to code for CD19t (OV19t), which produced a non-signaling, truncated CD19 for expression at the cell surface upon infection of cells [73]. Following the delivery of CD19-CAR T cells, OV19t was shown to promote tumor control and local immunity. Further, via the CAR T cell-mediated lysis of cancer cells, virus release was enhanced, which in turn amplified the expression of CD19 on more tumor cells, ultimately increasing the overall therapeutic efficacy.

Pancreatic ductal adenocarcinoma (PDA) also has an immunosuppressive TME. Clinical trials for anti-mesothelin CAR T cells for PDA have shown limited success. By combining anti-mesothelin CAR T cells with a cytokine-armed adenovirus, Watanabe et al. observed improved antitumor efficacy, enhanced T cell infiltration, significant tumor regression, and alteration in host tumor immunity in murine PDA models [174]. Notably, the study used an adenovirus expressing murine TNF-α and IL-2, as well as mouse CAR T cells. These two molecules are possible adjuncts to increasing an effective immune response beyond CAR T and OV-related changes.

Nishio et al. treated subjects with the oncolytic adenovirus Ad5Δ24n equipped with the chemokine RANTES and cytokine IL-15 in combination with GD2-CAR T cells in a neuroblastoma model [175]. Ad5Δ24 induced robust tumor cytotoxicity via the acceleration of the caspase pathway. Meanwhile, the release of RANTES and IL-15 from tumor cells promoted the recruitment and survival of CAR T cells, ultimately prolonging the survival of treated mice.

A further synergistic effect was exhibited via the local treatment of head and neck squamous cell carcinoma by systemic HER2-CAR T cell infusion and treatment with the adenovirus CAd12_PDL1 [176]. Survival in the xenograft models improved to over 100 days compared to around 25 days for mice treated by either approach alone. The growths of primary and metastasized tumors were also controlled in orthotopic models in the same study.

Proinflammatory cytokines produced by CAR T cells can increase T cell checkpoint signals, including PDL1, which can hinder CAR T cell function. By modifying an adenovirus to express a PD-L1 blocking mini-antibody, Tanoue et al. produced CAd-VECPDL1 to enhance the function of HER2-specific CAR T cells [177]. When administered on its own, helper-dependent adenovirus encoding the PD-L1 blocking mini-body, did not exhibit antitumor effect. Likewise, tumor reduction by the modified OV CAd-VECPDL1 was found to be comparable to that by parental OV. However, a combination of CAd-VECPDL1 with HER2-specific CAR T cells resulted in higher therapeutic efficacy compared to either these cells alone or combined with parental OV. 

Combination therapy is not without its restrictions, as demonstrated by Evgin et al. They observed that an mIFNβ-encoding oncolytic VSV (VSVmIFNβ) induced a strong inflammatory response, as expected [178]. Contrary to expectation, however, the remodeled TME did not potentiate CAR T cell delivery or function. Instead, the authors observed attrition of EGFRvIII CAR T cells, the mechanism for which is unknown. 

There is a scarcity of clinical trials specifically combining OVs with CAR-T cells for solid tumors, with only one trial underway. This human phase 1 study aims to investigate the effect of a binary oncolytic adenovirus and HER-2 specific CAR T cells in advanced HER2+ solid tumors, including GC [177].

## 5. Future Perspectives 

CAR T cell and OV combination therapy is a promising, early-phase strategy in the treatment of solid tumors, such as GC. However, many questions remain unanswered. Firstly, the optimal sequence, timing, and route of delivery must be established. The OV is typically administered first followed by CAR T cells. However, the optimal length of time that should elapse between virus and CAR T cell delivery for the most effective results must be determined for each agent and combination. This may vary based on the type of OV, route of delivery that affects the time required to reach its tumor target, rate of viral infection and replication that determine tumor kill, and any additional receptors or molecules that may be encoded by the OVs. 

The route of delivery is a major area of study in the OV-CAR T cell combination approach where logistical aspects must be considered. The most common and well-established drug delivery method in treating patients with solid tumors, including GCPC, is the IV systemic administration of anti-cancer agents. However, while IV administration is routinely performed and systemically delivered OV/CAR-T cells are more likely to reach dispersed metastases in peritoneal tumors, this is suboptimal in GCPC and other cancers with peritoneal spread, as the blood-peritoneal barriers limit peritoneal tumor penetration via systemic delivery. Moreover, OVs may be neutralized by antibodies in the bloodstream in immunocompetent individuals who may eventually build resistance as OV treatment continues. Thus, additional pre-conditioning may be required to prime patients for optimal combination therapy outcomes. Another option is IT administration, which can be more challenging, particularly for non-superficial lesions. IT administration advantageously allows for the safe and direct delivery of greater amounts of virus compared to the IV route without significant systemic toxicities [179]. Preclinical studies that we have reviewed here have demonstrated that the IP route of administration is superior in safely and effectively delivering OVs to directly target peritoneal tumors. IP administration benefits from both evading systemic immunity and delivering higher dosages and larger volumes. IP OVs in combination with either IV or IP CAR T cell combinations should continue to be explored and may become the preferred methods for the treatment of GCPC patients. 

Finally, the identification of the most effective tumor-specific molecular targets and selection of the right OVs and CAR T cell combinations suitable for treating GCPC will require continued preclinical and clinical research. Modifications to OVs or CAR T cells to enhance anti-tumor immune function independently or boost synergistic effect can improve both their safety and efficacy. The consideration of multi-target synchronous CAR T cell or OV administration may also be worthwhile. As noted by Shaw and Suzuki [180], the majority of preclinical studies for CAR T cell and OV therapy involved immunodeficient murine models. An improvement in the in vivo models for the testing of combination therapy can be pursued to identify clinically relevant effects or barriers when undertaking preliminary studies prior to proceeding to clinical trials. 

## 6. Conclusions

The field of precision oncology has made tremendous progress in translating molecular targets into clinically effective immunotherapeutic strategies against solid tumors. However, we have only begun to shift the survival curve, and hope for prolonged survival of GCPC patients remains fleeting. Alone and in combination, CAR T cells and OVs show exciting promise against GCPC, which largely remain refractory to other treatment modalities such as chemoradiation, monoclonal antibodies, and surgery. When combined, OVs and CAR T cells can achieve greater anti-tumoral efficacy with limited toxicity. Future evolutions of CAR T cells and OVs should be explored as adaptive immunotherapeutic strategies for advanced GC and GCPC. Novel OVs and CAR T cell combinations against GC may yield a more sophisticated immunotherapeutic strategy not only for GCPC patients but for many other difficult-to-treat solid tumors.

## Figures and Tables

**Table 1:** Preclinical studies in CAR-T for GC.

Author (Year)	Target	In Vitro	In Vivo	Dosing	Results
Chi et al. (2019) [32]	CEA	Human gastric (MGC803), pancreatic (AsPC1, BxPC3, PANC1), and colorectal (HT29) cancer cell lines	Xenograft subcutaneous gastric, pancreatic, and colorectal cancer nude mice models (*n* = 16 per cancer type, 4 cancers) 4 groups within cancer type: Control, control + rhIL-12 at days 7, 9, 12, 15, 19, and 25, CEA-CAR-T at day 7, CEA-CAR-T + rhIL-12	In vitro: 2 × 10^4^ CAR-T cells alone (2:1 effector:target ratio) 1 × 10^5^ CAR-T cells in combination with rhIL-12 In vivo: 5 × 10^6^ CAR-T cells IV day 0, 1 × 10^7^ CAR-T cells IV 1500 U/mouse rhIL-12	CEA-CAR-T alone treatment with significant reduction in tumor burden in vivo for all cancer types Combination of rhIL-12 with CEA-CAR-T enhanced anti-tumor efficacy of CEA-CAR-T cells significantly (*p* < 0.01) rhIL-12 significantly increased serum IL-2, TNF-α, and IFN-γ levels and significantly increased CAR-T proliferation in vitro
Jiang et al. (2019) [31]	CLDN18.2	Normal gastric (*n* = 24 types) and primary GC (*n* = 75 types) cell panels Patient-derived GC cells (GA0006, GA0060)	Xenograft and patient-derived xenograft (PDX) subcutaneous GC mice (*n* = 12 per model) 3 groups in cancer cell xenograft model (*n* = 6 per group): untransduced T cells, hu8E5-28Z CAR-T, hu8E5-2I-28Z CAR-T at tumor size 100 mm^3^ 2 groups in PDX model (*n* = 6–7 per PDX model group): untransduced T cells, hu8E5-2I-28Z CAR-T cells at tumor size 100 mm^3^	In vitro: 3:1, 1:1, and 1:3 effector:target ratio In vivo: 1 × 10^7^ CAR-T cells IV	Highly selective binding of hu8E5 and hu8E5-21 to CLDN18.2 Effective CLDN18.2-positive specific cell lysis by hu8E5-28Z and hu85-2I-28Z CAR-T cells in vitro with increased cytokine production Reduction in tumor volume in cancer cell-derived and patient-derived xenograft tumor models with hu8E5-28Z CAR-T treatment compared to untransduced T cell treatment (*p* < 0.01 for both models) Significant infiltration and persistence of CLDN18.2-specific CAR-T cells in CLDN18.2 positive tumors
Han et al. (2018) [33]	HER2	Human GC cell lines (NCI-N87, HGC27, MKN45, BGC-823, MKN28), ovarian cancer cell line (SKOV3)	Xenograft subcutaneous GC and intraperitoneal GCPC NSG mice models (*n* = 12 per model) 2 groups per model (*n* = 6 per group): chA21-4-1BBz CAR-T, untransduced T cells	In vitro: 1:1, 3:1, 10:1, 30:1 effector:target ratio In vivo: Subcutaneous GC—1 × 10^7^ CAR-T cells at days 40 and 45 after tumor inoculation GCPC—3 × 10^6^ CAR-T on day 0 and 1 × 10^7^ CAR-T cells IP on day 7 and day 10	Suppression of GCPC development with significant reduction in ascites, tumor nodule development, and prolonged survival with chA21-4-1BBz CAR-T treatment (*p* = 0.0005) Regression of tumor volume in HER2 overexpressing NCI-N87 model with CAR-T treatment (*p* < 0.0001) but progressive tumor growth in HER2 low-expressing MKN28 model regardless of treatment group demonstrating antigen-specific tumor elimination chA21-4-1BBz CAR-T cell population expansion in response to stimulation of high expression of target HER2 antigen cell lines
Jung et al. (2020) [34]	ICAM	Human GC cell lines (SNU719, NCC24, SNU638, SNU1, SNU5, SNU601, MKN28, Hs746t)	Xenograft systemic and GCPC NSG mice models Systemic (*n* = 4–6 per group): No T, NT, ICAM-1 CAR-T at day 8 after tumor inoculation CAR-T administration route GCPC model (*n* = 3 per group, 7 groups): No T cells, non-transduced T cells, or CAR-T cells at low or high doses either IV or IP at day 5 after tumor inoculation Combination therapy GCPC model (*n* = 5 per group, 6 groups): No T cells, non-transduced T cells alone, CAR-T alone, paclitaxel alone, non-transduced T cells + paclitaxel, CAR-T + paclitaxel	In vitro: 2.5:1 effector:target ratio In vivo: Systemic—10 × 10^6^ CAR-T cells IV GCPC only: Low dose 1 × 10^6^ CAR-T cells, high dose 10 × 10^6^ CAR-T cells IV or IP Combined paclitaxel dosage—1 × 10^6^ CAR-T cells IP IL12 group dosage—15 × 10^6^ cells IP	ICAM-1 expression level correlates with efficacy of ICAM-1 CAR-T cell Addition of IL12 (*p* < 0.0001) or paclitaxel (*p* < 0.01) therapy with CAR-T augments anti-tumor CAR-T activity in GCPC IP delivery of CAR-T cell therapy is more efficacious than IV delivery (*p* < 0.05) Although minimal toxicity of CAR-T cell treated mice, some mice developed tumor relapse or graft-versus-host disease
Cao et al. (2021) [35]	Mesothelin (MSLN)	Human GC (N87, MKN28, AGS), liver cancer (Huh-7) cell lines	Xenograft and patient-derived subcutaneous GC and GCPC NSG mice models 3 groups in subcutaneous GC (*n* = 5): PBS, MSLN-CAR NK, CD19-CAR NK at 50 mm^3^ tumor volume with treatment weekly, 3× treatment 3 groups in GCPC model (*n* = 5 per group): PBS, MSLN-CAR NK, CD19-CAR NK at days 10, 15, 20, and 25	In vitro: 16:1, 8:1, 4:1, 2:1 effector:target ratio In vivo: 5 × 10^6^ IV for subcutaneous GC model or IP for GCPC model	MSLN highly expressed in most gastric cancers but not in normal gastric tissue or other cancers Significant reduction in tumor weight in subcutaneous GC model with MSLN-CAR NK (0.23 g) treatment compared to control (1.22 g) and CD19-CAR NK (1.06 g) groups (*p* < 0.01) and also in PDX subcutaneous GC models (MSLN-CAR NK vs. control *p* < 0.001, MSLN-CAR NK vs. CD19-CAR NK *p* < 0.01) Increased survival, decreased bioluminescence in GCPC with MSLN-CAR NK than control or CD19-CAR NK groups (*p* < 0.01)
Lv et al. (2019) [36]	Mesothelin	Human GC cell lines (AGS, BGC-823, Kato III, MKN28)	Xenograft subcutaneous GC and GCPC NSG mice models 5 groups in subcutaneous model (group size not specified): No T cells, GFP-T IV, GFP-T peri-tumorally, M28z10-T IV, M28z10-T peri-tumorally when tumors are palpable 3 groups in GCPC model (*n* = 5 per group): No T cells, GFP-T, M28z10-T	In vitro: 2:1, 1:1, 1:2, 1:4 effector:target ratio In vivo: Subcutaneous model—5 × 10^6^ CAR-T cells IV or peri-tumorally GCPC model—5 × 10^6^ CAR-T cells IV	Higher levels of GC cell line cytotoxicity and cytokine secretion (IL-2, Granzyme B, GM-CSF, IFN-γ) with M28z10-T CAR-T cell treatment compared to GFP-transduced T cells (*p* < 0.001) Peritumoral injection of M28z10 CAR-T cells most efficacious in reduction in tumor volume and T cell infiltration compared to IV- or IT-delivered GFP-transduced T cells or IV M28z10 T cells (*p* < 0.01)
Zhao et al. (2021) [37]	Mesothelin	Human GC cell lines (BGC-823, MKN28, Kato III, MKN45)	Xenograft subcutaneous GC NSG mice models (2 cell line models) 3 groups per cell line model: Mock T cell injection (*n* = 4), anti-MSLN-T cells (*n* = 6), anti-MSLN-sP (*n* = 5, co-expression of sPH20-IgG2 with anti-MSLN CAR) when tumors palpable	In vitro: 1:1, 1:2, 1:4 effector:target ratio In vivo: 5 × 10^6^ CAR-T cells IV	Hyaluronic acid synthases interfere with anti-MSLN CAR-T cells via restriction of CAR-T cell mobility, are seen in multiple solid tumors, and are associated with worse outcomes Enhanced GC tumor growth inhibition (*p* < 0.01) and cell infiltration (*p* < 0.05) with added secretion of human hyaluronidase PH20 with anti-mesothelin CAR-T cells
Tao et al. (2018) [38]	NKG2DL	Human gastric cancer cell lines (MKN28, SNU1, SGC7901, MKN45), normal cells (HMEC1, GES1, THLE3)	Xenograft subcutaneous GC NSG mice models (*n* = 18) 3 groups (*n* = 6 per group): NKG2D CAR-T, mock-transduced T cells, PBS	In vitro: 2.5:1, 5:1, 10:1 effector:target ratio In vivo: 5 × 10^6^ CAR-T cells IV	Enhanced NKG2D CAR-T cell cytotoxicity compared to mock transduced T cells across all GC cell lines at 10:1 and most cell lines above 5:1 effector:target ratios (*p* < 0.001) Increased GC cell susceptibility to CAR-T cells with cisplatin pre-treatment via upregulation of NKG2D ligand expression in GC cells
Zhao et al. (2019) [39]	PD-L1	Human gastric cancer cell lines (BGC823, MGC803)	Xenograft subcutaneous GC NSG mice models (*n* = 20) 4 groups (*n* = 5 per group): untreated, CD19 CAR-T, Trop2 CAR-T, Trop2/PD-L1 CAR-T cells at day 14, 18, 22, and 26 after tumor inoculation	In vitro: 2:1, 5:1, 10:1, 20:1 effector:target ratio In vivo: 1 × 10^7^ T cells IT	Increased IL-2, IFN-γ production by bi-specific Trop2/PD-L1 CAR-T cells in Trop2^+^, PD-L1^+^ cells compared to control effector cells Tumor growth significantly inhibited by Trop2/PD-L1 CAR-T cells compared to CD19 or Trop2 CAR-T treatment (*p* < 0.05)

GC: gastric cancer; CAR-T: chimeric antigen receptor T cells.

**Table 2:** Clinical Trials in CAR-T for GC and GCPC.

Trial # (Year)	Phase of Trial	Inclusion Criteria	Target	Dosing	Route of Treatment	Status
NCT05396300 (2022)	1	Patients with CEA-positive advanced malignant solid tumors (colorectal, esophageal, stomach, pancreatic, metastatic, recurrent)	CEA	3–10 × 10^6^ CAR-T cells/kg	IV or IP	Recruiting
NCT05415475 (2022)	1	Patients with CEA-positive advanced malignant tumors (colorectal, esophageal, stomach, pancreatic, metastatic, recurrent)	CEA	1–10 × 10^7^ CAR-T cells/kg	IV or IP	Recruiting
NCT04348643 (2020)	1, 2	Patients with relapsed/refractory CEA-positive cancer (lung, colorectal, liver, gastric, pancreatic, breast)	CEA	Not specified	IV	Recruiting
NCT02349724 (2015)	1	Patients with relapsed or refractory CEA-positive malignant solid tumors (lung, gastric, breast, pancreatic, colorectal)	CEA	Not specified	Not specified	Recruiting
NCT05275062 (2022)	1	Patients with CLDN18.2-positive advanced gastric/esophagogastric cancer that failed at least second-line therapy or advanced pancreatic cancer that failed at least first-line therapy	IM92	2.5 × 10^8^ CAR-T cells	Not specified	Recruiting
NCT04864821 (2021)	1	Patients with CD276-positive solid tumors (osteosarcoma, neuroblastoma, gastric cancer, lung cancer)	CD276	Not specified	IV or IT	Not yet recruiting
NCT04427449 (2020)	1, 2	Patients with CD44v6-positive cancers	CD44	1 × 10^6^ CAR-T cells/kg	IV	Recruiting
NCT03874897 (2019)	1	Patients with CLDN18.2-positive solid tumors that failed standard systemic treatment	CLDN18.2	2.5 × 10^8^, 3.75 × 10^8^ or 5.0 × 10^8^	IV	Recruiting
NCT05277987 (2022)	1	Patients with CLDN18.2-positive advanced gastric/esophagogastric junction and pancreatic adenocarcinoma	CLDN18.2	Doses: 1st—0.5 × 10^6^ CAR-T cells/kg 2nd—0.5 × 10^6.5^ CAR-T cells/kg 3rd—0.5 × 10^7^ CAR-T cells/kg	Not specified	Recruiting
NCT03159819 (2017)	1	Patients with CLDN18.2-positive advanced gastric adenocarcinoma that failed first-line treatment and pancreatic adenocarcinoma refractory to surgical intervention or first-line systemic treatment	CLDN18.2	Not specified	IV	Recruiting
NCT03890198 (2019)	1	Patients with CLDN18.2-positive unresectable gastric adenocarcinoma or advanced pancreatic ductal carcinoma	CLDN18.2	Not specified	IV	Terminated
NCT02862028 (2016)	1, 2	Patients with EGFR-family-positive advanced solid tumors (lung, gastric, liver)	EGFR+	1–5 × 10^7^ CAR-T cells/kg	IV	Recruiting
NCT03563326 (2018)	1	Patients with EpCAM-positive advanced gastric cancer with peritoneal metastasis	EpCAM	Not specified	IP	Recruiting
NCT05028933 (2021)	1	Patients with malignant tumors of the digestive system (gastric, colorectal, liver, pancreatic)	EpCAM	Doses: 1st—3 × 10^5^ CAR-T cells/kg 2nd—1 × 10^6^ CAR-T cells/kg 3rd—3 × 10^6^ CAR-T cells/kg	IV	Recruiting
NCT04151186 (2019)	N/A	Patients with refractory/recurrent advanced pancreatic, colorectal, gastric, or lung cancer	EpCAM	Doses: 1st—2–2.5 × 10^5^ CAR-T cells/kg 2nd—4–5 × 10^6^ CAR-T cells/kg 3rd—8–10 × 10^6^ CAR-T cells/kg	IV	Not yet recruiting
NCT02725125 (2016)	2	Patients with recurrent/refractory stomach cancer	EpCAM	100 mL/time, 5 times	Not specified	Recruiting
NCT04650451 (2020)	1, 2	Patients with HER2+ solid tumors (gastric, breast, etc.)	HER2	Not specified	IV	Recruiting
NCT04660929 (2020)	1	Patients with HER2 overexpressing solid tumors	HER2	Group 1: Dose escalation Day 1—≤5 × 10^8^ cells Day 3—≤1.5 × 10^9^ cells Day 5—≤3 × 10^9^ cells Group 2: Full dose day 1 ≤ 5 × 10^9^ cells	IV, IP	Recruiting
NCT04511871 (2020)	1	Patients with recurrent/refractory HER2+ solid tumors	HER2	Dose cohorts: Dose 1—3 × 10^5^ CCT303-406 CAR-T cells/kg Dose 2—1 × 10^6^ CCT303-406 CAR-T cells/kg Dose 3—3 × 10^6^ CCT303-406 CAR-T cells/kg Dose 4—1 × 10^7^ CCT303-406 CAR-T cells/kg	IV	Recruiting
NCT03740256 (2018)	1	Patients with HER2-positive solid tumors	HER2	Dose cohorts: Level 1—CAdVEC: 5 × 10^9^ PFU, HER2 CAR-T cells: 0 Level 2—CAdVEC: 1 × 10^10^ PFU, HER2 CAR-T cells: 0 Level 3—CAdVEC: 1 × 10^10^ PFU, HER2 CAR-T cells: 1 × 10^6^ Level 4—CAdVEC: 1 × 10^11^ PFU, HER2 CAR-T cells: 1 × 10^6^ Level 5—CAdVEC: 1 × 10^11^ PFU, HER2 CAR-T cells: 1 × 10^7^ Level 6—CAdVEC: 1 × 10^12^ PFU, HER2 CAR-T cells: 1 × 10^7^ Level 7—CAdVEC: 1 × 10^12^ PFU, HER2 CAR-T cells: 1 × 10^8^	IT	Recruiting
NCT02617134 (2015)	1, 2	Patients with MUC1+ malignant glioma of the brain, colorectal carcinoma, and gastric carcinoma	MUC1	Not specified	Not specified	Recruiting
NCT02839954 (2016)	1, 2	Patients with MUC1-positive recurrent/refractory solid tumors	MUC1	Not specified	Not specified	Unknown
NCT05239143 (2022)	1	Patients with advanced or metastatic epithelial-derived solid tumors	MUC1	3 + 3 design of dose-escalating cohorts of single and multiple doses, dosages not specified	IV	Recruiting
NCT05166070 (2021)	1	Patients with recurrent/refractory MSLN-positive solid tumors	Mesothelin	Doses: Group 1—1.0 × 10^6^ CAR-T cells/kg Group 2—3.0 × 10^6^ CAR-T cells/kg Group 1—6.0 × 10^6^ CAR-T cells/kg	IV	Recruiting
NCT05141253 (2021)	1	Patients with recurrent/refractory MSLN-positive solid tumors	Mesothelin	Doses: Group 1—1.0 × 10^6^ CAR-T cells/kg Group 2—3.0 × 10^6^ CAR-T cells/kg Group 1—6.0 × 10^6^ CAR-T cells/kg	IV	Recruiting
NCT03054298 (2017)	1	Patients with mesothelin expressing cancers	Mesothelin	Dose cohorts: Group 1—1–3 × 10^7^ CAR-T cells/m^2^ Group 2—1–3 × 10^7^ CAR-T cells/m^2^ plus 1 g/mm^2^ cyclophosphamide Group 3—1–3 × 10^8^ CAR-T cells/m^2^ Group 4—1–3 × 10^8^ CAR-T cells/m^2^ plus 1 g/mm^2^ cyclophosphamide Group 5—1–3 × 10^7^ CAR-T cells/m^2^ intrapleural Group 6—1–3 × 10^7^ CAR-T cells/m^2^ plus 1 g/mm^2^ cyclophosphamide, then up to 2× additional CAR-T infusions Group 7—1–3 × 10^7^ CAR-T cells/m^2^ IP plus lymphodepleting chemotherapy plus up to 2× additional CAR-T infusions	IV, IP, and intrapleural infusion	Recruiting
NCT03615313 (2018)	1, 2	Patients with MSLN-positive advanced recurrent/refractory malignant solid tumors	Mesothelin	Not specified, PD-1 antibody-expressing CAR-Ts	IV	Recruiting
NCT03182803 (2017)	1, 2	Patients with MSLN-positive advanced recurrent/refractory malignant solid tumors	Mesothelin	2–5 × 10^7^ CTLA-4 and PD-1 antibody-expressing CAR-T cells/kg	IV	Recruiting
NCT04981691 (2021)	1	Patients with MSLN-positive advanced solid tumors that have failed at least first-line or second-line therapy	Mesothelin	Dose cohorts: 3 + 3 dose escalation Group 1—1 × 10^9^ CAR-T cells/infusion Group 2—3 × 10^9^ CAR-T cells/infusion	IV	Recruiting
NCT04107142 (2019)	1	Patients with recurrent/refractory solid tumors (colorectal, triple-negative breast, sarcoma, nasopharyngeal, prostate, gastric)	NKG2DL	Dose cohorts: 3 + 3 dose escalation Group 1—3 × 10^8^ CAR-T cells/infusion Group 2—1 × 10^9^ CAR-T cells/infusion Group 3—3 × 10^9^ CAR-T cells/infusion	IV	Not yet recruiting
NCT04847466 (2021)	2	Patients with advanced gastric/gastroesophageal junction cancers or head and neck cancers who failed standard treatment	PD-L1	2 × 10^9^ CAR-T cells/infusion	IV	Recruiting
NCT03960060 (2019)	1	Patients with recurrent/refractory stage IV metastatic solid tumors (soft tissue sarcoma, gastric, pancreatic, bladder cancer)	ROR2	Dose cohorts: 3 + 3 dose escalation Group 1—1 × 10^6^ CAR-T cells/kg/infusion Group 2—3 × 10^6^ CAR-T cells/kg/infusion Group 3—1 × 10^7^ CAR-T cells/kg/infusion	IV	Active, not recruiting

IV: intravenous; IP: intraperitoneal; IT: intratumoral; CAR-T: chimeric antigen receptor T cells.

**Table 4:** Clinical trials in oncolytic viruses for GC and PCGC.

Trial # (Year)	Phase of Trial	Inclusion Criteria	Treatment	Dosing	Route of Treatment	Status
NCT01443260 (2011)	1, 2	Patients with advanced peritoneal carcinomatosis or peritoneal mesothelioma	GL-ONC1	Every 4 weeks up to 4 cycles via infusion at 3 doses: 1 × 10^7^, 1 × 10^8^, 1 × 10^9^ PFU	IP	Completed (*n* = 9, 24 doses given)
NCT00794131 (2008)	1	Patients with advanced solid tumors	GL-ONC1	28 day cycle and 3 + 3 dose escalation 1 × 10^5^, 1 × 10^6^, 1 × 10^7^, 1 × 10^8^, 1 × 10^9^, 3 × 10^9^ PFU on day 1, 1.667 × 10^7^, 1.667 × 10^8^, 1.667 × 10^9^ pfu on days 1–3	IV	Completed (*n* = 43)
NCT03866525 (2019)	1, 2	Patients with malignant solid tumors (gastrointestinal cancers, head and neck cancers, soft tissue sarcomas)	OH2 (HSV OV) with or without irinotecan or HX008 (anti-PD-1 antibody)	Phase 1 dose escalation: three doses (1 × 10^6^, 1 × 10^7^, 1 × 10^8^ CCID_50_/mL) Phase 2 dose expansion: OH2 single agent vs. OH2 + irinotecan vs. OH2 + HX008	IT	Recruiting

IP: intraperitoneal; IV: intravenous; IT: intratumoral; CCID_50_: cell culture infectious dose 50%.

## Data Availability

Not applicable.
